# Genome-Wide Identification and Expression Analysis of *SNAP* Gene Family in Wheat

**DOI:** 10.3390/genes15101311

**Published:** 2024-10-11

**Authors:** Xiaohan Zhang, Yanan Yu, Yumeng Sun, Yan Bai, Yongjun Shu, Changhong Guo

**Affiliations:** Key Laboratory of Molecular Cytogenetics and Genetic Breeding of Heilongjiang Province, College of Life Science and Technology, Harbin Normal University, Harbin 150025, China; zxhan2024@163.com (X.Z.); yynan2024@163.com (Y.Y.); sym19283746@163.com (Y.S.); kaku3008@hrbnu.edu.cn (C.G.)

**Keywords:** *Triticum aestivum* L., *SNAP* gene family, abiotic stress, expression analysis

## Abstract

**Background/Objectives**: The *SNAP* gene family is a class of proteins containing a SNAP domain, which plays a crucial role in the growth and development of plants. **Methods**: Bioinformatics methods were used to systematically analyze the gene structure, phylogenetic evolution, chromosomal distribution, physicochemical properties, conserved motifs, and cis-acting elements of the *TaSNAP* family members. **Results**: The *TaSNAP* family comprises members that encode proteins ranging between 120 and 276 amino acids, with isoelectric points spanning from 4.87 to 7.92. Phylogenetic analysis elucidated the categorization of the eight *TaSNAP* into three distinct subfamilies, wherein members of the same subfamily display marked similarities in their gene structures. Chromosomal mapping revealed the distribution of *TaSNAP* family members across chromosomes 2A, 2B, 2D, 7A, 7B, and 7D. Utilizing the Plant CARE tool, we identified ten elements linked to plant hormones and four associated with stress responses. Expression analysis via qRT-PCR was performed to assess the levels of the eight *TaSNAP* genes in various tissues and under diverse abiotic stress conditions. The results indicated heightened expression of most genes in roots compared to spikes. Notably, under ABA stress, the majority of genes exhibited upregulation, whereas certain genes were downregulated under PEG stress, implying a substantial role for SNAP protein in wheat growth and development. **Conclusions**: This study conducted a comprehensive bioinformatics analysis of each member of the wheat *SNAP* family, laying a crucial foundation for future functional investigations.

## 1. Introduction

Wheat (*Triticum aestivum* L.) is a plant in the Poaceae family and belongs to the genus *Triticum*. As an important food crop [1], it has an extremely wide cultivation area and a profound socio-economic impact worldwide. The cultivation area accounts for about 1/3 of the arable land, and approximately 35% to 40% of the global population relies on it as their staple food [2]. The production and quality of wheat are influenced by numerous environmental pressures throughout its growth and developmental stages, among which salinity [3], drought conditions [4], and low temperatures [5] stand out as the most significant abiotic stressors. Wheat is a moderately salt-tolerant plant, and when the soil salinity exceeds a certain threshold, wheat growth is inhibited. This can lead to stunted growth and development and, in severe cases, plant death [6]. Saline soils are widely distributed in China, ranging from tropical to cold temperate zones, from coastal to inland areas, and from humid regions to extremely arid desert areas. The total area of saline soils in China is about 3.6 million hectares, accounting for 4.88% of the country’s usable land area [7]. The main concentrated distribution areas of saline soils in China are the northwest, north, and northeast regions and coastal areas. Among them, the saline soil area of the six western provinces and regions (Shaanxi, Gansu, Ningxia, Qinghai, Inner Mongolia, and Xinjiang) accounts for 69.03% of the national total [8]. Under the influence of global warming, drought events are becoming more frequent, with increasing intensity and duration, leading to progressively severe impacts. Over the past half-century, the boundary between semi-arid and semi-humid areas in China has generally moved southward, and the area of drought has been increasing [9]. In particular, drought conditions are more severe in Xinjiang, the Tibetan Plateau, and the southeast regions, while most areas of Central and East China are in a state of light drought or no drought. Therefore, research on salt and drought stresses is of great importance for improving wheat yield and quality. According to previous studies on SNAP proteins in plants, SNAP proteins are involved in various plant stress responses, and *SNAP* can be expressed under salt stress induction, showing higher tolerance to salt stress in plants [10]. SNAP proteins have been reported in many plants, but so far, no systematic analysis of the wheat *SNAP* gene family has been conducted. Therefore, this study analyzes the *SNAP* gene family based on the wheat whole genome, providing a reference for further research on the functions of this gene family. The results of this study are of great significance for further breeding or improving wheat varieties with resistance.

SNARE proteins (Soluble NSF Attachment Protein Receptor) are a class of key proteins involved in membrane fusion. They play a crucial role in processes such as intracellular material transport, secretion, and endocytosis. SNARE proteins can be divided into v-SNAREs (located on vesicle membranes) and t-SNAREs (located on target membranes, including members of the Syntaxin family and *SNAP-25*) [11]. *SNAP* (Synaptosome-Associated Protein) is a type of t-SNARE that, together with Syntaxin, forms part of the SNARE complex, which plays a central role in processes such as neurotransmitter release. The *SNAP* family is a protein family with a SNAP domain, also known as the Soluble NSF Attachment Protein Receptor (SNARE) gene family [11], which is widely present in eukaryotic cells. These proteins play a key role in vesicle transport and membrane fusion within the cell [12].

In plant cells, vesicle transport is a key process for maintaining cell function and responding to environmental challenges. Especially under abiotic stresses such as salt stress, active vesicle transport is crucial for maintaining cellular homeostasis [13]. SNARE proteins, as regulators of vesicle transport, ensure the accurate delivery of substances to target organelles by mediating membrane fusion. Currently, the *SNAP* gene family has been reported in potatoes, tomatoes, rice, and *Arabidopsis*, and in the study of potatoes, it has been shown that *StSNAP* genes are mainly related to vesicle transport. By studying the *StSNAP30* gene of ‘Qinghu No. 9’ potatoes, various bioinformatics methods have been used to analyze the structural characteristics, physiological and biochemical properties, gene expression patterns, and subcellular localization of StSNAP30 protein [14]. This has verified the biological function of *StSNAP30* in the development of potato pollen and pollen tube growth. In *Arabidopsis*, it has been found that *AtSNAP33* is a t-SNARE protein homologous to *SNAP-25*, which works with KEULE and KNOLLE proteins in the process of cell division and cell plate formation, promoting vesicle fusion. Mutations in the *AtSNAP33* gene can lead to plant growth and development defects and affect the plant’s response to pathogens [15]. In addition, the expression of *AtSNAP33* is regulated by salicylic acid (SA), indicating that it may play an important role in the plant’s immune response. Subsequently, scientists have discovered a gene encoding a Qbc-SNARE protein, *SlSNAP33.2*, in tomatoes, which is induced under salt stress. Transgenic tomato plants overexpressing *SlSNAP33.2* show improved salt stress tolerance, which is related to the promotion of endocytosis and the accumulation of sodium ions in vacuoles [16]. This endocytosis helps maintain cellular ion balance and reduce the accumulation of hydrogen peroxide, thus protecting cells from damage caused by salt stress. These studies indicate that *AtSNAP33* and *SlSNAP33.2*, and other *SNAP25*-type proteins, play an important role in the response of plants to biotic and abiotic stresses. Not only do they participate in the modulation of cell division and exocytosis but also they potentially contribute to the regulation of plant immune responses and stress resilience as well. Improving the expression or function of these proteins through genetic improvement may help cultivate crop varieties that are more tolerant to salinity, drought, or other abiotic stresses [17,18].

Salt stress has a significant impact on the growth, development, yield, and quality of wheat [19]. As an important food crop, wheat productivity is severely limited by abiotic stresses such as soil salinization. Studies have shown that salt stress can cause inhibited wheat growth, yellowing of leaves, and inhibited root growth, and ultimately it can affect overall growth, development, and yield performance [20].

Under salt stress conditions, the physiological response of wheat includes a decrease in osmotic potential and disturbance of ion balance, leading to water deficiency and cellular homeostasis imbalance, which may eventually cause cell death. Wheat activates a variety of physiological and biochemical mechanisms to adapt to salt stress, including osmotic regulation, photosynthesis and respiration metabolism, hormone distribution, ion distribution, and reactive oxygen species (ROS) scavenging [21].

Utilizing wheat whole-genome data retrieved from the Ensembl Plants database [22], this study focuses on the identification of *SNAP* gene family members. It proceeds to examine their physicochemical attributes, gene architecture, conserved motifs, chromosomal localization, cis-regulatory elements, and phylogenetic evolution [23]. Additionally, qRT-PCR analysis was conducted to elucidate the expression profiles of eight *SNAP* family genes in various tissues and under diverse abiotic stress conditions, thereby establishing a foundation for an enhanced comprehension of the functions and significance of wheat SNAP proteins [24].

## 2. Materials and Methods

### 2.1. Plant Material

Chinese Spring wheat (*T. aestivum* L.) served as the experimental material for this study. The wheat seeds (provided by the Key Laboratory of Molecular Cytogenetics and Genetic Breeding of Heilongjiang Province, Harbin, China) underwent a disinfection process involving immersion in 75% alcohol for a duration of 3 min. This was followed by three rinses with distilled water and a subsequent soak in 10% sodium hypochlorite for another 3 min. After thorough rinsing with distilled water, the seeds were placed in sterile Petri dishes to facilitate germination and were cultivated for a period of 24 h. Upon sprouting, the seeds were transplanted into hydroponic boxes filled with 1/2 Hoagland nutrient solution and maintained in a greenhouse under constant temperature conditions and a 16 h photoperiod. At the two-week stage of wheat seedling development, root, stem, and leaf tissues were harvested for tissue expression analysis. Subsequently, stress treatments were applied using 0.2 mol/L NaCl (sourced from Hu Shi), 20% PEG (supplied by Solarbio, Beijing, China), 100 μmol/L ABA (from CMBIO, Pennsylvania, PA, USA), and exposure to 4 °C, with plants grown under normal conditions serving as controls. These controls were maintained under optimal temperature, sufficient light, appropriate water supply, without the addition of any exogenous hormones, and in soil devoid of stress-inducing substances. Following a 6 h treatment period, root, stem, and leaf tissues were collected from both treated and control plants, with each sample being replicated three times. Additionally, wheat spikes and pollen, collected from field-grown plants between April and July, were subjected to tissue expression analysis. Each sample of these tissues was also replicated three times. All collected samples were promptly frozen using aluminum foil, stored at −80 °C, and reserved for subsequent RNA extraction procedures.

### 2.2. Identification and Physicochemical Property Analysis of Wheat SNAP Gene Family Members

The whole-genome data of wheat, along with its protein sequences and annotation files, were retrieved from the Ensembl Plants database (http://plants.ensembl.org/index.html, accessed on 8 May 2024). The hidden Markov model (HMM) representing the *SNAP* family member structure domain was obtained from the Pfam database (http://pfam.xfam.org/, accessed on 8 May 2024). Utilizing HMMER 3.0 software in conjunction with this *SNAP*-specific HMM, a search was conducted through the wheat genome’s functional protein sequence database to identify potential wheat SNAP protein sequences. To validate the authenticity of these predicted genes as *SNAP* family members, the wheat SNAP protein sequences, after removing duplicates, were subjected to protein structure prediction using both SMART (http://smart.embl-heidelberg.de/, accessed on 8 May 2024) and NCBI’s Conserved Domain Database (CDD) online tool. This was performed to ascertain the presence of the conserved domain unique to *SNAP*, termed *TaSNAP*. Additionally, the ExPASy website (https://web.expasy.org/protparam/, accessed on 8 May 2024) was employed to assess fundamental physicochemical properties of the wheat SNAP protein sequence, including molecular weight, isoelectric point, and stability.

### 2.3. Systematic Evolutionary Analysis of Wheat SNAP Gene Family

For the multiple sequence alignment of wheat *SNAP* amino acid sequences, ClustalX software (version 2.1) was employed. Subsequently, a phylogenetic tree was constructed using MEGA software (version 11.0.13), applying the Neighbor-Joining algorithm. The parameters for this analysis included Poisson correction, pairwise deletion, and a bootstrap value of 1000 repetitions (the website usage time was May 2024).

### 2.4. Gene Structure and Conserved Motif Analysis of Wheat SNAP Gene Family

The GSDS website was used to draw the intron and exon gene pattern diagram, and the intron and exon information was downloaded from the Plant Transcription Factor Database (http://planttfdb.cbi.pku.edu.cn/, accessed on 10 May 2024) and SGN (https://solgenomics.net/, accessed on 10 May 2024). The online software MEME (https://meme-suite.org/meme/tools/meme, accessed on 10 May 2024) was used to predict conserved motifs.

### 2.5. Chromosome Localization Analysis of Wheat SNAP Gene Family

The chromosome position of *TaSNAP* was extracted from the wheat gene information GFF3 file, and the online website MG2C (http://mg2c.iask.in/mg2c_v2.1/, accessed on 10 May 2024) was used to make a chromosome localization map.

### 2.6. Cis-Acting Element Analysis of Wheat SNAP Gene Family

The promoter region (upstream 2000 bp) of each wheat *SNAP* gene was extracted from the wheat whole-genome database, and Plant CARE software (https://bioinformatics.psb.ugent.be/webtools/plantcare/html/, accessed on 11 May 2024) was used to analyze cis-acting elements related to plant hormones and stress responses.

### 2.7. RNA Extraction and Real-Time Fluorescence Quantitative PCR Analysis

The Primer 5 software was used to design specific quantitative primers (see Table 1), with wheat Actin as the reference gene. The extraction of wheat total RNA was accomplished utilizing an RNAprep Pure Plant Kit, followed by the conversion of the isolated RNA into cDNA employing a TransStart^®^Top Green qPCR SuperMix (AQ131, TRAN) reagent. The composition of the PCR reaction mixture is outlined below: 2 μL of cDNA, 10 μL of 2× TransStart^®^Top Green qPCR SuperMix, 0.4 μL each of the forward and reverse primers, and 7.2 μL of nuclease-free water. The amplification program is as follows: 94 °C for 30 s, 94 °C for 5 s, 54 °C for 15 s, 72 ° C for 31 s, 40 cycles. Each sample was performed in triplicate, and the data were processed using the 2^−ΔΔCT^ method. R (version 4.2.2) was used for difference significance analysis, with *p* < 0.05 indicating significant difference and *p* < 0.01 indicating extremely significant difference.

## 3. Results

### 3.1. Basic Information Analysis of Wheat SNAP Gene Family Members

HMMER 3.0 software was utilized to obtain the amino acid sequences of wheat *SNAP* genes. Subsequently, protein structure predictions were conducted using SMART and NCBI’s online tool CDD to verify that the wheat *SNAP* genes contain the conserved domain unique to *SNAP*, resulting in the identification of eight wheat *SNAP* gene family members. These members were numbered from top to bottom based on their relative positions on the chromosome and named *TaSNAP1* to *TaSNAP8* (Table 2). These *TaSNAP* gene family members encode 120 to 276 amino acids, with isoelectric points ranging from 4.87 to 7.92 and molecular weights ranging from 31782.45 to 37561.43 Da. Among them, seven are acidic proteins (isoelectric point < 7.0) and one is a basic protein (isoelectric point > 7.0).

### 3.2. Systematic Evolutionary Analysis of Wheat SNAP Gene Family Members

**Figure 1:**
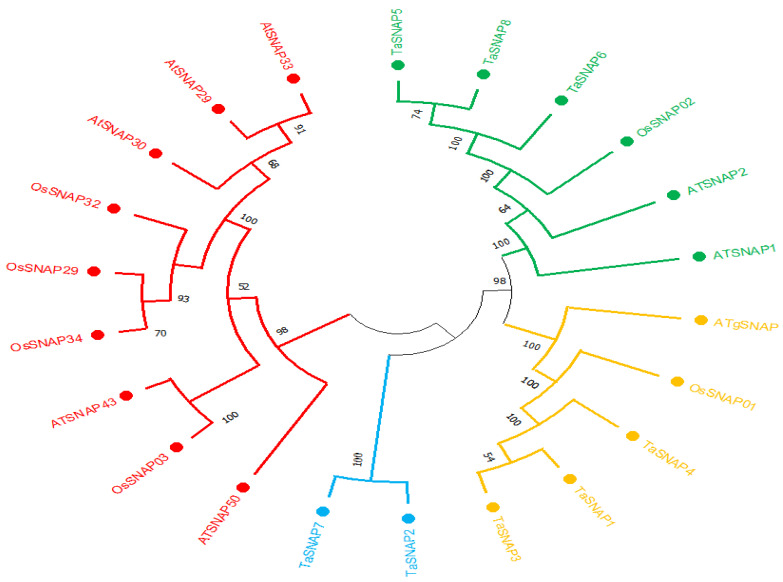
The unrooted phylogenetic tree of *SNAP* gene family in wheat and the phylogenetic tree of *SNAP* proteins in *Arabidopsis*, wheat, and rice. The NJ tree was constructed using the TaSNAP amino acid sequence with MEGA software (version 11.0.13) and 1000 bootstrap repetitions. Wheat SNAP proteins were divided into three groups (Group I marked with blue solid circles, Group II marked with yellow solid circles, and Group III marked with green solid circles; red solid circles do not contain wheat SNAP proteins, so they were not included in the grouping). The numbers at the nodes are bootstrap values, representing support levels.

MEGA 7.0 software was used to compare the *SNAP* protein sequences of known model plants *Arabidopsis* and rice with the SNAP protein sequences of wheat to construct a *SNAP* phylogenetic tree (Figure 1). As shown in Figure 1, based on the wheat *TaSNAP* gene structure, they were divided into three subfamilies. Subfamily I members are *TaSNAP2* and *TaSNAP7*, subfamily II members are *TaSNAP1*, *TaSNAP3*, and *TaSNAP4*, and subfamily III members are *TaSNAP5*, *TaSNAP6*, and *TaSNAP8*. It is speculated that members of the same subfamily may have the same function.

### 3.3. Structural Feature Analysis of Wheat SNAP Gene Family

**Figure 2:**
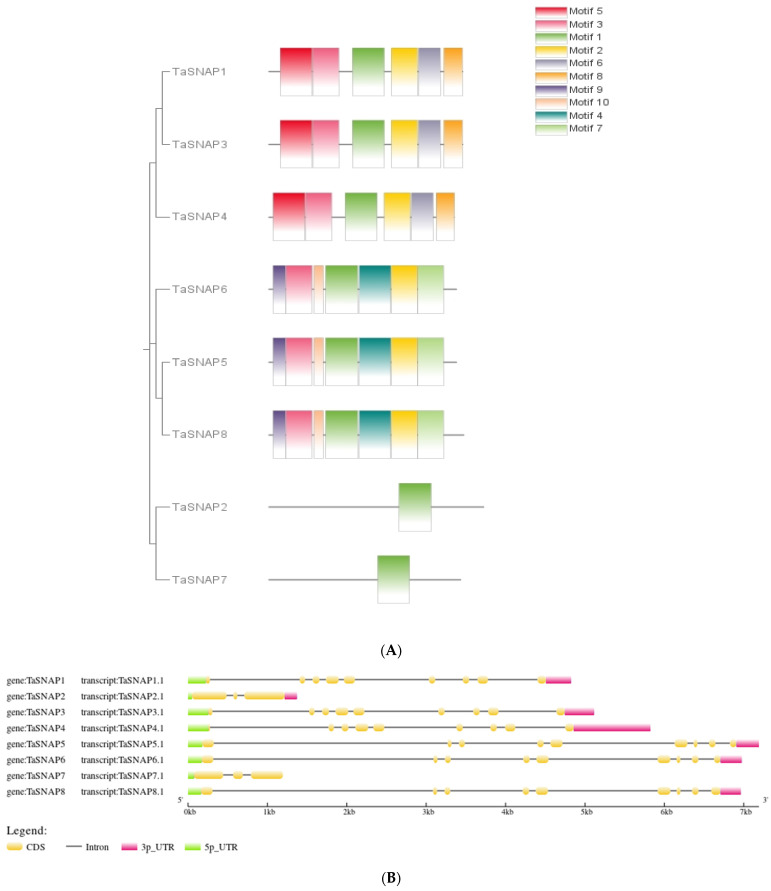

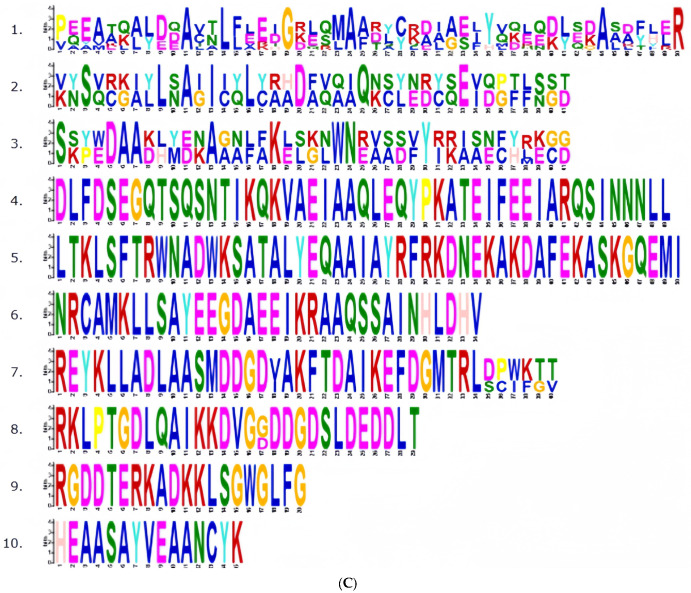
(**A**) presents the phylogenetic and gene structure analysis of *TaSNAP*, while (**B**) shows the protein structure analysis of *TaSNAP*. Through the results, the grouping and function of *TaSNAP* are analyzed. (**A**): Phylogenetic tree; gene structure; (**B**): protein structure; (**C**): conserved domains of B-box genes family in wheat.

As shown in Figure 2, except for subfamily III members, the structures of subfamily I and subfamily II members are relatively similar, and subfamily III members only contain three exons. The exon count remains consistent across members of subfamily I and subfamily II, with each comprising precisely nine exons, indicating a robust uniformity in their gene structures within the respective subfamilies. Furthermore, the gene architecture of these *SNAP* genes bears resemblance to that observed in *Arabidopsis* and rice.

In order to analyze the structural characteristics of the wheat *SNAP* family, 10 conserved motifs in the wheat *SNAP* gene protein sequence were predicted using the online tool MEME (Figure 2), namely, Motif 1, Motif 2, Motif 3, Motif 4, Motif 5, Motif 6, Motif 7, Motif 8, Motif 9, and Motif 10. As shown in the figure, all members of subfamily I contain Motif 1, Motif 2, Motif 3, Motif 5, Motif 6, and Motif 8; all members of subfamily II contain Motif 1, Motif 2, Motif 3, Motif 4, Motif 7, Motif 9, and Motif 10; subfamily III members only contain Motif 1, and it is found that Motif 1 exists in all members, while Motif 5, Motif 6, and Motif 8 are only present in subfamily I members, and Motif 4, Motif 7, Motif 9, and Motif 10 are only present in subfamily II members. This indicates that the wheat *SNAP* gene family may have lost or gained conserved motifs during the process of evolution.

### 3.4. Chromosome Localization Analysis of Wheat SNAP Gene Family

**Figure 3:**
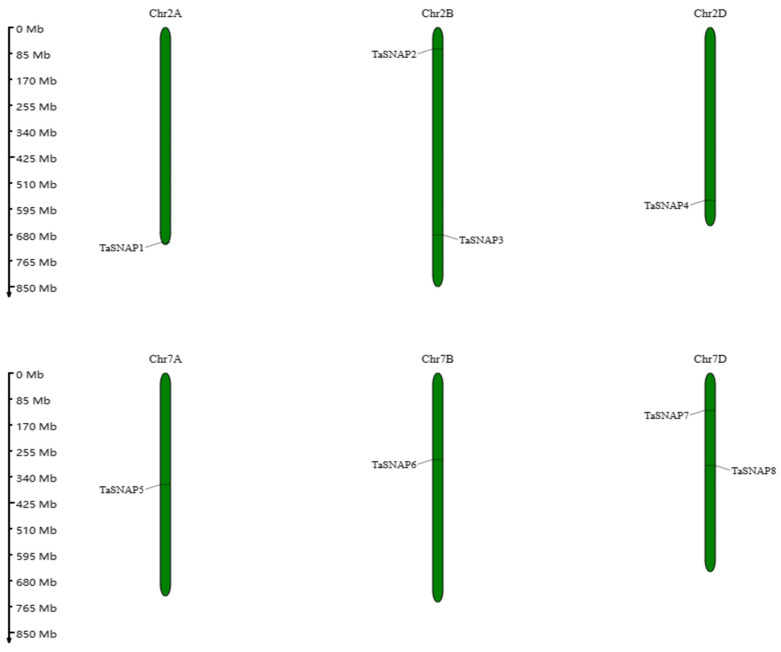
The chromosome location of the *SNAP* gene family in wheat.

As shown in Figure 3, the eight wheat *SNAP* genes are unevenly distributed on six chromosomes, with two genes on chromosomes 2B and 7D and one gene on each of the other four chromosomes.

### 3.5. Cis-Acting Element Analysis of Wheat SNAP Gene Family

**Figure 4:**
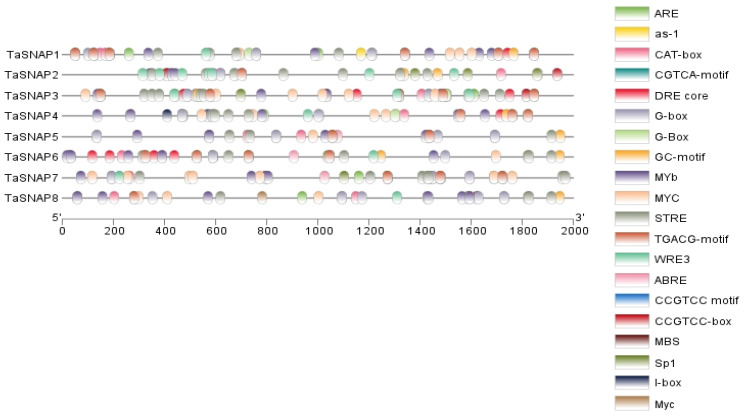
Cis-acting element analysis of *TaSNAP*.

To gain insight into the potential regulatory mechanisms governing the expression of wheat *SNAP* gene family members, we identified the cis-acting elements within eight wheat *SNAP* genes. Our analysis revealed a total of 20 such elements linked to plant hormones and stress responses (depicted in Figure 4). Notably, numerous elements are associated with stress responses, encompassing the DRE core (Dehydration Responsive Element), MYB, STRE (Stress Responsive Element), CCGTCC motif, and other stress-related elements. These elements are integral to various plant physiological processes, including responses to low temperatures, high salinity, and drought stress. The DRE core element is particularly pivotal in plant stress responses, as DREB transcription factors bind to it to modulate the expression of specific genes, thereby facilitating plant adaptation to adverse environmental conditions. Research conducted by the Fujian Agriculture and Forestry University team on Moso bamboo highlighted the significance of the *PeDREB28* gene in enhancing plant tolerance to abiotic stress [25]. DREB transcription factors are known to regulate the expression of genes related to stress responses such as drought, salt, and cold tolerance. Harnessing DRE cis-acting elements and DREB transcription factors can bolster plant stress resistance [26]. Moreover, exploring the interplay between DRE cis-acting elements and DREB transcription factors is crucial for elucidating the molecular mechanisms underlying plant stress signal transduction [27]. Our analysis of the diverse cis-acting elements identified in *TaSNAP*, along with their interactions and functions, underscores the importance of the *SNAP* gene family in plants. Given the presence of various stress-related elements within *SNAP*, it plays distinct roles in different abiotic stresses, enhancing plant cold and drought resistance capabilities.

### 3.6. Expression of Wheat SNAP Gene Family in Different Tissues and Abiotic Stresses and Hormone Treatments

**Figure 5:**
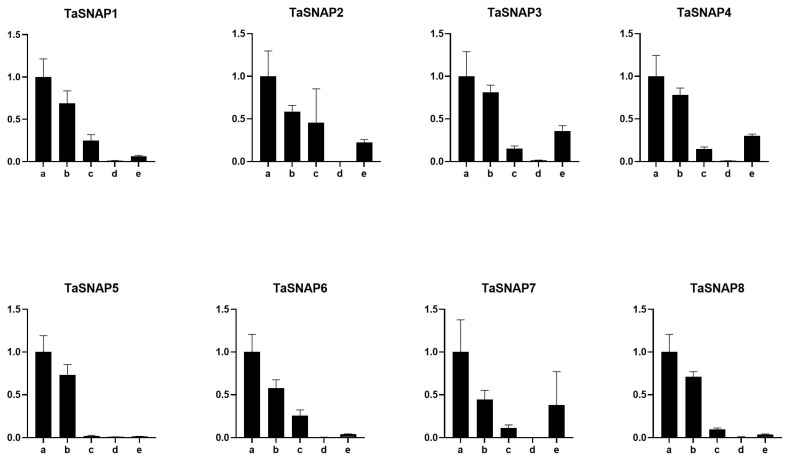
Expression profile of *TaSNAP* gene family in different tissues of wheat. a: root; b: stem; c: leaf; d: spike; e: pollen.

**Figure 6:**
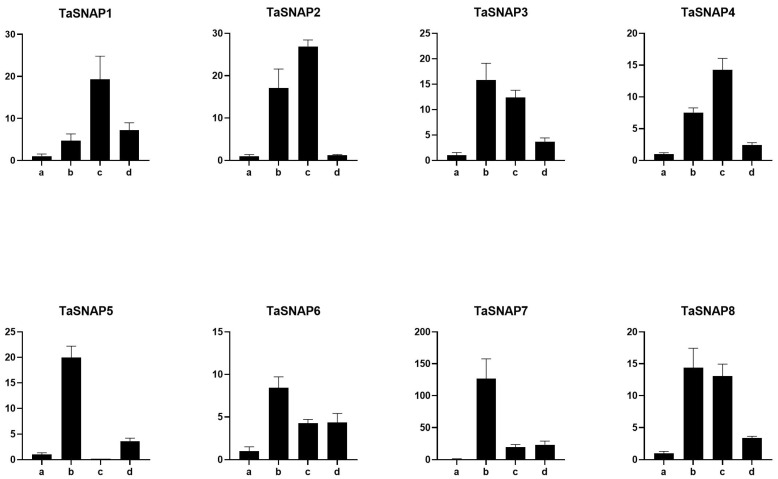
Expression profile of *SNAP* gene family under different treatments in wheat. Note: a: control; b: six hours of 0.2 mol L^−1^ NaCl treatment; c: six hours of 100 μmol L^−1^ ABA treatment; d: six hours of 20% PEG treatment.

qRT-PCR was employed to investigate the expression profiles of *TaSNAP* across various tissues, including roots, stems, leaves, spikes, and pollen, as well as in response to diverse abiotic stress conditions such as osmotic stress, low temperature, salinity, and abscisic acid (ABA) treatment (refer to Figure 5 and Figure 6). The eight genes exhibited differential expression across all tested tissues, with roots demonstrating the highest expression levels and spikes showing the lowest. Specifically, *TaSNAP5* displayed minimal expression in leaves, spikes, and pollen. Within the *SNAP* gene family, a consistent expression pattern emerged across tissues, with roots consistently exhibiting the peak expression, followed by stems. However, notable variations in expression were observed among leaves, spikes, and pollen.

*TaSNAP* responds to abiotic stress, but the expression levels are different (Figure 6). Under salt stress treatment, the expression of the *SNAP* family genes was upregulated, but the upregulation levels were different among different family members. Among the *TaSNAP3*, *TaSNAP5*, *TaSNAP6*, *TaSNAP7*, and *TaSNAP8* family members, the upregulation levels were extremely significant, and these five members had higher salt tolerance compared to other members. Under osmotic stress, *TaSNAP1*, *TaSNAP3*, *TaSNAP4*, *TaSNAP5*, *TaSNAP6*, *TaSNAP7*, and *TaSNAP8* were all upregulated. Except for the *TaSNAP5* gene, the other seven family members were upregulated under ABA stress. In the eight genes of the *SNAP* family, the expression trend in different tissues was roughly the same, and only the *TaSNAP5* gene was different. Through qRT-PCR analysis of the expression patterns of *TaSNAP* in different tissues (roots, stems, leaves, spikes, pollen) and abiotic stress treatments (osmotic, low temperature, salt, and ABA), further analysis of the *TaSNAP* gene family revealed that the highest expression was in roots, which also represents the strongest salt and osmotic resistance in roots compared to other tissues, and each family member responded to salt stress and osmotic stress.

## 4. Discussion

In plant cells, substance transport is crucial for maintaining cell function and responding to environmental challenges. SNARE proteins (Soluble N-ethylmaleimide-Sensitive Factor Attachment Protein Receptor) play a key role in the fusion of cell membranes, promoting the fusion of vesicles with target membranes by forming specific protein complexes [27]. SNARE proteins play a variety of important roles in the cell, including participating in the formation of cell plates as sites for the synthesis of new cell walls [28], regulating vesicle fusion to control hormone release, and mediating vesicle transport to regulate the secretion of defense-related proteins and compounds, thereby responding to pathogen attacks [29].

The *SNAP* family is a family of proteins with a *SNAP* domain, also known as Soluble NSF Attachment Protein. SNARE proteins can be divided into v-SNARE and t-SNARE. *SNAP* (Synaptosome-Associated Protein) [30] is a type of t-SNARE that, together with Syntaxin, forms part of the SNARE complex, which plays a central role in processes such as neurotransmitter release. *SNAP* is widely present in eukaryotic cells and plays a key role in intracellular vesicle transport and membrane fusion. In different plant species, members of the *SNAP* gene family play important roles in pollen development, cell division, and responses to biotic and abiotic stresses [31].

Research results indicate that the *SNAP* gene family plays a key role in the growth and development of plants, as well as in stress response [32]. For instance, proteins such as *Arabidopsis* AtSNAP33, tomato’s SlSNAP33.2, rice’s OsSNAP32, and potato’s StSNAP30 exhibit diverse biological functions in vesicle transport, cell division, pollen development, and responses to salt stress and pathogen attacks. Overexpression of the *SlSNAP33.2* gene in tomato enhances the plant’s tolerance to salt stress. This is achieved by promoting endocytosis and the accumulation of sodium ions in vacuoles, thereby reducing the accumulation of hydrogen peroxide in the cytoplasm of root hair cells, indicating that SlSNAP33.2 plays a significant role in the endocytic pathway of plants in response to salt stress [14,15,16,17,18].

This study identified eight wheat *SNAP* family members and conducted bioinformatics analysis, revealing that these members have similar physicochemical properties, with the number of amino acids ranging from 120 to 276 and the isoelectric point (pI) values between four and eight. The chromosomal localization analysis of these genes shows that they are relatively evenly distributed across six chromosomes (2A, 2B, 2D, 7A, 7B, 7D). A phylogenetic analysis of *SNAP* proteins from wheat, rice, and *Arabidopsis* was conducted, and based on the gene structure of the *SNAP* family members, these family members were divided into three subfamilies. Members within the same subfamily have similar structures and the same number of exons, suggesting that they may have similar functions [33].

Cis-acting elements are non-coding DNA segments identified within the promoter region of genes [34]. The analysis of these elements among the *TaSNAP* gene family members has revealed numerous elements associated with plant hormones and responses to abiotic stresses, including abscisic acid (ABA), ethylene, gibberellins, auxins, low temperature, and drought [35]. Among them, the abscisic acid-responsive element-binding proteins (AREBs/ABFs) can bind to the abscisic acid-responsive element (ABRE) and participate in the response to ABA, dehydration, and high salinity stresses.

qRT-PCR was used to analyze the expression patterns of *TaSNAP* under different tissues and abiotic stress treatments [36]. All eight genes were expressed to varying degrees in all tested tissues, with the highest expression in roots, followed by stems, and varying expression in leaves, spikes, and pollen. This suggests that the salt and drought tolerance of roots is superior to that of other tissues. At the same time, *TaSNAP* responds to abiotic stresses, but the expression levels vary under different stresses. Under salt stress treatment, the expression of the *SNAP* family genes was upregulated; under drought stress, *TaSNAP1*, *TaSNAP3*, *TaSNAP4*, *TaSNAP5*, *TaSNAP6*, *TaSNAP7*, and *TaSNAP8* were significantly upregulated; except for the *TaSNAP5* gene, the other seven genes were upregulated under ABA stress.

Based on the research, it is speculated that the differential expression of the *SNAP* gene family is related to the response to environmental stresses. For example, the *SNAP* genes play a role in the response to biotic and abiotic stresses, and different family members have different expression levels under different stresses. The changes in their expression may also be related to the plant’s ability to adapt to different environmental conditions. In summary, this study provides the identification and analysis of wheat *SNAP* gene family members, including their physicochemical properties, phylogeny, gene structure, chromosomal location, cis-acting elements, and expression patterns, laying a theoretical foundation for further exploration of the role of this gene family in plant stress responses.

## 5. Conclusions

This study provides the identification and analysis of wheat SNAP gene family members, including their physicochemical properties, phylogeny, gene structure, chromosomal localization, cis-acting elements, and expression patterns. It is speculated that the differential expression of the SNAP gene family is related to the response to environmental stresses, and different family members have varying expression levels under different stresses. The changes in their expression may also be associated with the plant’s ability to adapt to different environmental conditions, providing a theoretical foundation for further exploration of the role of this gene family in plant stress responses.

## Figures and Tables

**Table 1:** Primers for quantitative real-time PCR of *TaSNAP* family.

Gene Name	Forward Primer (5′–3′)	Reverse Primer (5′–3′)
*TaSNAP1*	GCCAAGGCCGACAAACTA	GCATCCCACGGTGATGAG
*TaSNAP2*	CCGACCTAGCCGAGTTCCA	ATCCCACGCACTTCCGTTT
*TaSNAP3*	GAAGAGTGCTACCGCCTTGT	GTCGGAAACTTCATTCCAGAGT
*TaSNAP4*	AGAGTGCTACCGCCTTGT	CATCAGAGGCAGGTTGTG
*TaSNAP5*	TATGTGGAAGCCGCAAAC	CGGCCCTTTCTAGGTAATC
*TaSNAP6*	CCGCCGACCTATACGATA	GCAGCCATGCTCAATCTAC
*TaSNAP7*	TGGAGTTAGCCGAGTTCTACATG	CCGGTGGAGTAGTTGAAAGGAA
*TaSNAP8*	CCGCCGACCTATACGATA	GCAGCCATGCTCAATCTAC

**Table 2:** *TaSNAP* gene information identified in the wheat genome.

Gene Name	Gene ID	Coded Amino Acids	Isoelectric Point	Molecular Weight	Length (aa)	Group
*TaSNAP1*	TraesCS2A02G461600.1	298	4.97	33083.88	899	II
*TaSNAP2*	TraesCS2B02G109300.1	330	7.92	37561.43	995	I
*TaSNAP3*	TraesCS2B02G483200.1	298	5.03	33025.84	899	II
*TaSNAP4*	TraesCS2D02G461500.1	286	5.08	31782.45	863	II
*TaSNAP5*	TraesCS7A02G292400.1	289	4.87	32473.27	872	III
*TaSNAP6*	TraesCS7B02G182700.1	289	4.87	32505.33	872	III
*TaSNAP7*	TraesCS7D02G170100.1	296	5.18	34045.13	893	I
*TaSNAP8*	TraesCS7D02G284600.1	301	4.87	33725.76	906	III

## Data Availability

The original contributions presented in the study are included in the article, further inquiries can be directed to the corresponding authors.
